# West Nile Virus and Related Flavivirus in European Wild Boar (*Sus scrofa*), Latium Region, Italy: A Retrospective Study

**DOI:** 10.3390/ani10030494

**Published:** 2020-03-16

**Authors:** Angela Petruccelli, Tiziana Zottola, Gianmarco Ferrara, Valentina Iovane, Cristina Di Russo, Ugo Pagnini, Serena Montagnaro

**Affiliations:** 1 Department of Veterinary Medicine and Animal Productions, University of Naples, 80137 Naples, Italy; angela.petru@hotmail.it (A.P.); jammaferrara@hotmail.it (G.F.); upagnini@unina.it (U.P.); 2 Experimental Zooprophylactic Institute of Lazio e Toscana Regions, Section of Latina, 04100 Latina, Italy; tiziana.zottola@izslt.it (T.Z.); cristina.dirusso@izslt.it (C.D.R.); 3 Department of Pharmacy, University of Salerno, Via Giovanni Paolo II 132, 84084 Fisciano (SA), Italy; vale.iovane@gmail.com

**Keywords:** European wild boar, flavivirus, *Sus scrofa*, retrospective study, West Nile virus

## Abstract

**Simple Summary:**

A retrospective study was carried out on the presence of Flavivirus antibodies in the European wild boar (*Sus scorfa*) in the Lazio region of Italy. 168 serum samples from European wild boars were tested by cELISA, virus neutralization test (VNT), and RT-PCR. All sera that had cELISA anti-Flavivirus antibodies were further examined by the VNT. Thirteen wild boars (7.73%) were positive for Flavivirus by cELISA, a single European wild boar serum was positive for VNT, with a neutralizing antibody titer 1/14. No samples resulted positive for Flavivirus by RT-PCR assay.

**Abstract:**

Background: A retrospective sero-survey for evidence of West Nile virus (WNV) infection in European wild boar (*Sus scorfa*) was conducted in the Latium region, Italy, on stored serum samples of the period November 2011 to January 2012. Methods: Sera were collected from 168 European wild boars and screened for antibodies to WNV and other Flaviviruses by competitive enzyme linked immunosorbent assay (cELISA). All sera positive for Flavivirus antibodies by cELISA were further examined by virus neutralization test (VNT). To test the presence of Flavivirus RNA in samples, an RT-PCR was performed using a pan-Flavivirus primers pair. Results: Thirteen wild boars (7.73%) were seropositive for Flaviviruses. The hemolysis of serum samples limited the interpretation of the VNT for 7 samples, confirming the presence of specific antibody against WNV in a single European wild boar serum sample. The presence of ELISA positive/VNT negative samples suggests the occurrence of non-neutralizing antibodies against WNV or other antigen-related Flaviviruses. No samples resulted positive for Flavivirus by RT-PCR assay. Conclusion: Although a moderately high percentage of animals with specific antibody for WNV has been detected in wild boar in other surveillance studies in Europe, this has not been reported previously in Italy. Together, these data indicate that European wild boar are exposed to WNV and/or other related-Flavivirus in central Italy and confirm the usefulness of wild ungulates, as suitable Flavivirus sentinels.

## 1. Introduction

Flaviviruses are single-stranded positive-sense RNA viruses (family Flaviviridae) classified in the Genus Flavivirus, which can be grouped into vector-borne (mosquito- and tick-borne) Flaviviruses and Flaviviruses with no known arthropod vector (NKV) [1]. Flaviviruses have a worldwide distribution, and a lot of them, such as the West Nile virus (WNV) are cause of zoonoses significant for public health. In the United States, the virus was first identified in 1999 [2], in Europe, large WNV outbreaks have been reported. The widest European outbreak happened in 1996 in Bucharest, Romania [3].

The first human encephalitis outbreak on the West Nile was identified in Camargue, France, the Mediterranean area in 1962 [4], in the 1990s other epidemics involved a relatively large number of human cases in Algeria, Tunisia, and Israel. Over recent years, in the Volgograd region in Russia, Hungary, Romania, and Greece, cases of WNV encephalitis have been reported [5]. In 1998, the first outbreak of WNV infection was described in horses in Tuscany, Italy [6]. In the summer of 2008, the virus reappeared in northeastern Italy, where it caused a neuroinvasive infection in horses in the Veneto and Emilia Romagna regions [7]. In the summer of 2008, by means of the activation of an extraordinary WNV surveillance program, 9 human cases of West Nile Invasive Disease (WNND) were reported [8,9], another 16 cases were described at the end of summer 2009. All these events were found in the regions of Emilia Romagna, Veneto, and Lombardia, in the wetlands adjacent to the Po river.

In order to control and monitor WNV circulation, a virological and serological surveillance program for WND has been implemented, across the country, by the Ministry of Health [8].

The veterinary surveillance program, which involved 15 Italian wetlands, chosen for the presence of many aquatic birds, including some migratory birds, aimed to detect WNV introduction [8]. In fact, the West Nile virus is maintained in nature in an enzootic transmission cycle that primarily involves mosquitoes (*Culex* spp.) and birds [10,11,12]. Humans, horses, and other mammals are incidental hosts because they generally develop an inadequate viremia to infect susceptible mosquitoes [13,14,15]. 

In Europe, anti-WNV antibodies have been found in several bird species, however, there is little information on mammals other than humans or horses, and the serological sign of WNV infection has been described in a few domestic and wild mammal species [13,14,15]. Wildlife mammals may be involved in the virus transmission cycle as they are naturally exposed to flavivirus infections.

Although several WNV surveys have been performed on birds, as far as we know, there are no studies concerning wild mammals in Italy. Hunter-harvested mammals, such as European wild boar, often are more accessible than wild birds and allow the collection of more abundant blood samples. Thus it has often been proposed to use mammals as alternative Flavivirus sentinels because contact detection would show exposure outside the enzootic bird cycle [14,15].

Regarding the European wild boar (*Sus scrofa*), it is possible to assume an analogous response to that of domestic pigs (*Sus scrofa*). Pigs develop low/short viremias and it is unlikely that they are amplifying hosts, but they can be useful as sentinels due to their serological reaction [16].

Flavivirus antibody prevalence has been previously reported in wild boar from the Czech Republic [17], feral swine in the USA [18], and Eurasian wild boar from Spain [19].

In Central Italy, to monitor the circulation of WNV and USUV, Coseddu et al. [20] conducted serological surveillance in wild rodents, however no other mammalian population in Italy has been tested serologically for evidence of Flavivirus infection. In 2009, in Italy, the WNV circulation spread and also involved the region of Lazio, where cases of WND have appeared in horses [21].

For these reasons, we have undertaken a retrospective investigation of a collection of serum samples stored in our laboratory (2010–2011), to determine the degree of exposure to Flavivirus of the European wild boar in central Italy.

## 2. Materials and Methods 

The study area (41°28′02″ N; 13°32′21″ E to 41°21′41″ N; 13°44′02″ E-19.374 ha), located near the Natural Park Aurunci, which is part of the Mediterranean ecosystem, partially overlaps with one of the surveillance zones identified according to the national veterinary surveillance program [22]. 

Serum samples from hunted wild boar were obtained by convenience sampling from animals harvested by a local hunting association, near the Natural Park Aurunci in the provinces of Latina and Frosinone, during the hunting seasons (October–January) between 2011 and 2012.

Approval from the ethics committee was not required since no live animals were used for this study. Animals were harvested by standard procedures for the hunting of wild boar. Harvested wild boar were carried to a central processing site for cleaning, dressing, and sampling procedures. We collected 1–3 cm^3^ of whole blood via heart puncture or cranial sinus puncture. Blood samples were centrifuged at 1100× *g* for 15 min and frozen at −80 °C until testing [23]. For each sample, we recorded the location, the date of collection, the sex, and the age of the animal, determined using dentition patterns, classifying them into two age classes: 0–24 months old (juvenile), 25–36 months old (sub-adult), and >36 months old (adults).

The presence of antibodies against WNV and closely related Flaviviruses, like USUV, was analyzed using a commercial competitive enzyme-linked-immunosorbent assay (ELISA ID Screen West Nile Competition Multi-Species (Innovative Diagnostics, Montpelier, France),) for detection of West Nile virus antibodies against the pr-E and pr-M envelope proteins containing an epitope common to all Flaviviruses according to the manufacturer’s protocol [24,25,26]. This Elisa test has a high cross-reactivity, thus it is suitable for the detection of a wide range of flaviviruses, although it has been designed for the diagnosis of WNV infection. The assay used plates pre-coated with WNV recombinant antigens and assessed the competition between antibodies present in the serum samples tested and a monoclonal anti-E antibody conjugated to horseradish peroxidase (HRP). These competitive ELISA kits detected essentially every Ig isotype but was primarily used to reveal IgG and was classified between the IgG detection tools.

The samples were analyzed twice and read at 450 nm. The ELISA was validated when the residual binding rations (S/N %) were calculated. Serum samples with S/N ratios equal to or less than 40% were considered positive; samples with ratios greater than 50% were considered negative. S/N values between 40% and 50% were doubtful.

To verify cELISA positive samples, neutralizing antibody titers to WNV were determined by a virus-neutralization test (VNT), according to Monaco et al. [22]. A serum sample was considered positive if the cytopathic effect (CPE) neutralization was greater than 90% at the lowest dilution (1:10). The serum titer represents the highest serum dilution capable of neutralizing over 90% of the CPE in cell culture. VNT titles greater than or equal to 1/10 were considered WNV specific. To confirm the presence of Flavivirus RNA in positive wild boar samples, an RT-PCR was performed using pan-Flavivirus primers, according to Johnson et al. [27]. No template and WNV field isolate positive samples were used in the RT-PCR assay as negative and positive controls, respectively. Nucleic Acid was purified from blood samples of cELISA positive wild boars using Quiazol Lysis Reagent (Quiagen, Venlo, The Nederlands) following the manufacturer’s instruction. The viral RNA was amplified using degenerate primers Flavi-For (sense) 5′-GCMATHTGGTWCATGTGG-3′ and Flavi-Rev (antisense) 5′-GTRTCCCAKCCDGCNGTRTC-3′ [27]; the amplicon from this primers pair was located toward the endo of NS5 coding sequence and produced of the expected size of 203 bp. To synthesize the first-strand cDNA, the Super script II reverse transcriptase (Life Technologies, Carlsbad, CA, USA) was used. The mixture was incubated at 42 °C for 50 min, followed by heating at 95 °C for 5 min. Amplifications were performed using 2.5 U Takara LA Taq (Takara BIO, Inc., Japan) with the following thermal cycle: Initial denaturation at 94 °C for 3 min, 30 cycles of 94 °C for 45 s, 50 °C for 30 s, and 72 °C for 90 s [27]. PCR reactions were performed in a T100 Thermal Cycler (Bio-Rad Laboratories, Hercules, CA, USAi). The amplification products were analyzed by 1.5% agarose gel electrophoresis in TBE buffer (89 mM Tris-borate, 2 mM EDTA, pH 8.2) by ChemiDoc gel scanner (Bio-Rad Laboratories, Hercules, CA, USA). 

The statistical significance within each class (age and gender) was evaluated using a chi-square test by MedCalc Statistical Software version 16.4.3 (MedCalc Software, Ostend, Belgium;). The variables (gender and age) associated with the presence of specific antibodies against Flaviviruses have been applied to binary logistic models using JMP Pro version 15.0.0 (SAS Institute Inc., Milano, Italy.). *p* < 0.05 was considered significant.

## 3. Results

**Figure 1:**
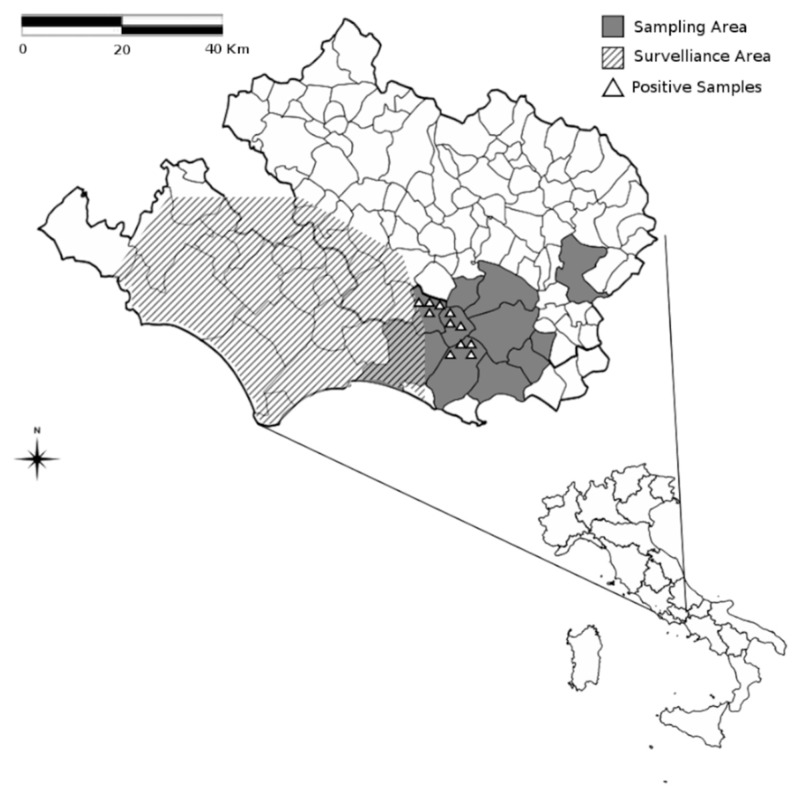
Geographic distribution of West Nile Disease virus/Flavivirus positive wild boars.

In this study, 168 wild boar serum samples were included. Figure 1 shows the area comprising the geographic distribution of West Nile Disease virus/Flavivirus positive wild boars. Overall, juveniles were the most represented category in the wild boar population (45%), followed by sub-adults (31%), and adults (24%). The sample consisted of 89 (53%) males and 79 (47%) females.

The overall cELISA Flavivirus positivity rate was 7.73% (13 out of 168, IC 95% 3,70–11,76). The univariate and multivariate analyses indicated that there was no association between gender and Flavivirus seropositivity (*p* > 0.05) while juvenile wild boars (13.15%, CI 95% 5.28–20.48) were significantly correlated with a risk of Flavivirus infection with an odds ratio of 7.72 (CI 95% 0.957–62.34) and 2.87 (CI 95% 0.599–13.83), respectively, vs. sub-adults and adults animals (Table 1).

The 13 cELISA positive samples were tested by VNT, however, the hemolysis limited the interpretation of the VNT for 7 samples, confirming the presence of WNV specific antibody only in a single European wild boar serum, with a 1/14 neutralizing antibody titer.

The 168 blood samples were further subjected to molecular detection of Flavivirus by RT-PCR using a pan Flavivirus primers pair [27]. No cELISA positive samples resulted positive for Flavivirus by RT-PCR assay.

## 4. Discussion

During our survey, 13 wild boars resulted positive for Flavivirus by cELISA, but VNT confirmed the presence of WNV specific antibody only in one European wild boar. ELISA positive, VNT negative samples indicate the presence of non-neutralizing antibodies versus WNV or against other antigenically related Flaviviruses, for example, the Usutu virus. In fact, even though Flavivirus activity has been limited in Italy, in the last decade, evidence of the circulation of Flaviviruses transmitted by mosquitoes such as WNV and Usutu virus (USUV) has been found with increasing frequency [28,29,30].

USUV has been circulating silently in Italy since 2007 [30]. The presence of this virus has been associated with fatal cases in birds, and the virus has been detected in the brain of immunodeficient patients with neuroinvasive infection [31,32]. Savini et al. [33] have shown the circulation of USUV in Italy, particularly in Tuscany, Veneto, Emilia Romagna, and Friuli. The presence of an endemic cycle in local species of birds and mosquitos is supported by the detection of the virus in wild birds and by seroconversion in horses and sentinel chickens [33].

Our study confirms the presence of Flavivirus infection (7.73%; 95% CI: 3.70–11.76) in the wild boar population of Latium Region.

Comparing to the prevalence found in other European countries, the values obtained in the wild boar in the present study are higher than those reported in the Czech Republic (4.1% ) [17], similar to that reported in France in 2009–2014 (5.6% for Flavivirus and 3.4% for USUV) [26,27,28,29,30,31,32,33,34], but are lower than those reported in Spain, with a mean prevalence of 12.6% but with a significantly higher seroprevalence (27%) in wetland habitats during 2006–2010 [19], and in Serbia by [34] (17.6% WNV prevalence). In this study, only four wild boar sera showed USUV specificity.

Furthermore, our results were also lower than those obtained in feral pigs in North America from ELISA, with an average prevalence of 22.5% in 2001—2004 [18].

Negative results obtained by molecular biology assay are similar to the results obtained by Gutierrez-Guzman et al. [19] in wild boar, red fox and other wild animals in 2012; by our group in hunting dogs [35], by Maggi et al. [29] and Lelli et al. [30] in human and horses respectively. In fact, viremia developed by human and other mammals infected by most of Flavivirus circulating in Europe is a low-level/short-lived viremia [36,37]. Indeed “dead-end” hosts like mammals, including humans, usually don’t represent amplifying hosts, but could be used as sentinels for their serological reaction [28,29,30,31,32,33,34,35,36,37,38,39].

Finally, the mismatch between molecular and serological results is probably attributable to the nature of assay used; in fact serology is the measure of historical exposure and may or may not be linked to viremia at the time of sampling [40], while RT-PCR is indicated to molecular confirmation of the infection but is not suitable for estimating the prevalence of the disease.

In our study, risk factor analysis did not detect any statistically significant effect (*p* = 0.3507) of gender, while there is a statistical correlation between flavivirus seropositivity and age (*p* = 0.049). Surprisingly we observed the highest presence of specific antibody in juvenile wild boar class with an odds ratio of 7.72 (CI 95% 0.957–62.34) and 2.87 (CI 95% 0.599–13.83) respectively Vs sub-adults and adults animals. This unexpected result is in contrast to previous results in North American feral swine [17] and in European wild boar in Spain [19]. Considering that in adult pigs, it has been previously demonstrated than antibodies to WNV may last for more than 3 years possibly due to frequent re-inoculation by mosquito bites [41] and that other experimental studies detected persistent antibodies in absence of re-infections until 28 days post infection [16,17,18,19,20,21,22,23,24,25,26,27,28,29,30,31,32,33,34,35,36,37,38,39,40,41,42], the negative relationship between WNV seropositivity and increasing age is consistent with epidemiological data of WNV transmission in Latium region [22] and suggest a recent time span of possible exposure and ongoing flavivirus transmission in the area. In fact, according to the national veterinary surveillance program’s data, WNV was detected in this area in 2009 but not in 2010 and 2011 [22]. 

These results can also be explained by the limited number of samples analyzed. However, convenience sampling is often the only one possible for assessing prevalence in wild animals and may be the only means to providing information quickly and cheaply [43].

Nevertheless, the presence of 12 cELISA positive samples and of one cELISA/VTN positive wild boar confirm the findings of Boadella et al. [44] about potential usefulness of juvenile wild ungulates, as suitable flavivirus sentinels.

The intensity of exposure to WNV in nature is very complex and depends on many factors such as the density of mosquito population, the mosquitoes behavior, the spatio-temporal distribution of the virus amplification hosts (birds) and their immunological status, and the availability of alternative sources of blood meals for the mosquitoes [8]. The results obtained in this study document the exposure of wild boars in central Italy to Flavivirus and provides evidence of the potential utility of European wild boar swine as sentinels of WNV. The advantages of using wild boar as WNV sentinels include: The opportunity to monitor WNV in a mammalian population (which may reflect human exposure more accurately than avian species), collaboration with already established wild boar collection programs (thus decreasing the cost of surveillance), the much larger quantities of serum available for testing than in mesomammals and small mammals, and the use of an overabundant feral species for surveillance rather than native fauna [18]. 

## 5. Conclusions

In conclusion, the results achieved in our work record the exposure of European wild boar to Flaviviruses in Italy, in an area where Flavivirus activity has previously been reported. This could indicate the transmission of Flavivirus outside the enzootic bird cycle. Further studies would be needed to evaluate Flavivirus circulation in other regions, and the degree of exposure of other widely distributed wild mammals would be of interest.

## Figures and Tables

**Table 1:** Presence of Flavivirus antibody and risk factor analysis in wild boar (*Sus scrofa*) in the Latium region (Hunting seasons 2011–2012) as detected by cELISA.

Factor	n	Flavivirus							
		n	%	SE%	95% CI	χ^2^	*p*-Value	OR	95% CI
Total	168	13	7.73	4.03	3.70–11.76				
Gender									
*Female*	79	4	5.06	4.83	0.23–9.89				
						0.871	*p* = 0.3507	0.47	0.140–1.604
*Male*	89	9	10.11	6.26	3.85–16.37				
Age									
*0–24 mounth (juvenile)*	76	10	13.15	7.33	5.28–20.48			Ref.	
*25–36 mounth (sub-adults)*	52	1	1.92	3.77	0–5.69	4.248	*p* = 0.0495	7.72	0.957–62.34
*>36 mounth (adults)*	40	2	5.0	6.75	0–11.75			2.87	0.599–13.83
